# Natural Spillover Risk and Disease Outbreaks: Is Over-Simplification Putting Public Health at Risk?

**DOI:** 10.1007/s44197-025-00412-y

**Published:** 2025-04-24

**Authors:** David Bell, Jean von Agris, Blagovesta Tacheva, Garrett Wallace Brown

**Affiliations:** 1 Independent Consultant, Lake Jackson, TX USA; 2 School of Politics and International Studies (POLIS), University of Leeds, Leeds, LS2 9JT UK

**Keywords:** Pandemic preparedness, PPPR, Zoonotic spillover, Outbreak risk, Public health policy

## Abstract

The pandemic prevention, preparedness and response (PPPR) agenda is currently dominating international public health. International agencies including the World Health Organization and World Bank are proposing an unprecedented level of funding that will inevitably have broad consequences across health and society. Arguments supporting pandemic policy are heavily based on the premise that pandemic risk is rapidly increasing, driven in particular by passage of pathogens from animal reservoirs to establish transmission in the human population; ‘zoonotic spillover’. Proposed drivers for increasing spillover are mostly based on environmental change attributed to anthropogenic origin, including deforestation, agricultural expansion and intensification, and changes in climate. Much of the literature, including reports published by international agencies and peer-reviewed papers, offers support for fundamental changes in public health policy premised on definitive statements that spillover is indeed increasing, that underlying anthropogenic drivers are the main reason for this, and that these are remediable. However, many of these assumptions are poorly supported by cited literature, over-simplifying a highly complex set of ecological interactions. This picture is further complicated by rapidly and unevenly evolving capacity for pathogen detection and notification. Public health policy based on incorrect assumptions and overly simplified analyses is likely to lead to poorly designed interventions and poor outcomes. If we are to deal effectively with outbreak risk within the broad context of competing public health priorities, there is an urgent need to re-evaluate current assumptions on drivers of outbreaks based on available evidence and address continuing major gaps in knowledge.

## Introduction

The current global pandemic prevention, preparation and response (PPPR) agenda is heavily premised on the potential for pathogens to ‘spillover’ from their predominant zoonotic hosts to humans [1–3]. This is widely considered a major driver of a reported increase in zoonotic outbreaks over the past fifty or more years, and also underpins the growing interest in One Health, under which investment in reducing human environmental impacts is proposed to mitigate disease risk [4]. Together, the World Health Organization (WHO) and World Bank propose over forty billion dollars be invested into PPPR and One Health to reduce overall risk. Return on this investment is therefore heavily dependent on zoonotic spillover being a major and increasing driver of risk, and that its mitigation will therefore help. Despite evidence that claims of more frequent and increasingly deadly pandemics are exaggerated [5, 6], the pandemic agenda remains a dominant theme in global health today, driving amendments to the recently revised International Health Regulations (IHR) and WHO’s proposed Pandemic Agreement [7, 8], both emphasizing increased surveillance for natural outbreaks.

Biology is complex, not least when it involves passage of pathogens between multiple species, often through intermediary vectors and dependent on specific human and non-human species behaviour. Complexity is increased when the environment within which these interactions occur is itself changing over time. Human population density (e.g. likelihood of transmission), prior exposure (e.g. immunity), and general population health (prevalence of risk factors such as obesity), and the availability of medical interventions further impacts outcomes. Perceptions of change are also influenced by diagnostic and communication changes that enable diseases to be distinguished, and funding to enable their use [9]. 

Disregarding this complexity with blanket statements regarding trends in natural zoonotic spillover events, and therefore future pandemic risk, poses considerable risk to public health due to the large resource diversions necessary to support the growing PPPR agenda. While simplification can sometimes be necessary in public messaging, oversimplification in policy development and promotion runs the risk of exacerbating harm. In this article, we review key claims about zoonosis spillover risk from two sources. First, we examine major reports published by international health agencies related to epidemic and pandemic preparedness. Second, we discuss key peer-reviewed publications which serve as an evidence base for these reports, and a number of highly cited articles that strongly back these arguments (e.g. Vora et al. [10]), implicate spillovers in pandemics or recognize a more complex and nuanced perspective (e.g. Gottdenker et al.’s review of anthropogenic impact [11]). Other aspects of the claims supporting increased PPPR investment warrant examination, including the veracity of the return on investment calculations themselves [12]. However, as the PPPR agenda of WHO is geared towards naturally-occurring outbreaks, it is essential to examine the robustness of the evidence on which these claims are being based before the fundamental shifts in international public health policy proposed by WHO can be justified.

## Risk Assertions of International Health Organizations

The WHO is currently expanding its role in pandemic preparation and management through recently accepted amendments to the International Health Regulations (IHR) and ongoing negotiations on the Pandemic Agreement [8, 13]. Both covenants repeat assertions about increasing pandemic risk, which are mirrored in the WHO’s 2023 revision of its handbook *Managing Epidemics*: [14]

Epidemics and pandemics of infectious diseases are occurring more often, and spreading faster and further than ever, in many different regions of the world.

While this claim is poorly founded [5, 6], it sets a tone of definitive statements that continues later when asserting that increased risk of zoonotic spillover events are a major driver of this perceived increase:

Deforestation, urban sprawl and human encroachment into previously untouched habitats intensify our interactions with wildlife and the pathogens they harbour. Changing and intensified food production, from live poultry and animal markets to deforestation for expanded large-scale agriculture, also leads to increased contact between people and wildlife. Some of the animals that humans are increasingly in contact with (bats for example) are likely sources of new pathogens.

In their 2022 report *Putting Pandemics Behind Us*, the World Bank recommend an additional $10.3 billion to $11.5 billion for One Health interventions, justified by claims of increasing spillover risk: [4]

Every year, zoonotic diseases sicken billions of people, killing millions, with low- and middle-income countries being most vulnerable.

and, as cause,

As humans extend their footprint on the planet, encroaching into natural habitats and altering them, the potential for diseases to emerge has increased exponentially.

The term ‘exponentially’ has a clear meaning, and this is a World Bank report, so one should reasonably assume that there is a strong basis for this assertion.

The Group of Twenty Nations (G20) Finance and Health Taskforce, comprised of WHO and the World Bank, submitted its 2022 report (with accompanying technical report) to the G20 to advocate for an urgent increase in funding for, and emphasis on, preventing and mitigating pandemics [3, 15]. To justify this position, the report makes bold statements regarding increased pandemic risk:

Outbreaks of infectious pathogens have been a defining feature of human history, and any analysis of prevailing trends strongly suggests that outbreaks of pathogens of pandemic potential are set to continue to increase in frequency for the foreseeable future." and later:

The intensification of, and interaction between, factors such as ecological degradation, climate change, conflict and resource competition, mass population movement and displacement, urbanization, global travel and trade, and changes in agricultural practices continue to multiply the risks of emergence and re-emergence of epidemic and pandemic threats.

A critical aspect of these statements is the strength of the Taskforce’s claims for the underlying mechanism driving this perceived risk: “…*any analysis* of prevailing trends strongly suggests…” and “[various listed anthropogenic changes] *continue to multiply* the risks…”. *Any analysis* is an unusual statement for a complex epidemiological phenomenon and strongly implies a fixed and indisputable set of causes and effects.

The High-Level Independent Panel (HLIP) of the G20, contributing to the same meeting in Bali, made similar assertions in its separate 2022 report: [2]

Scientists attribute the increased frequency of infectious disease outbreaks to population growth and increased human encroachment on the natural environment; the loss of the world’s biodiversity; the growth of the wildlife trade; increasing urbanization, crowded living conditions and increased mobility; and the broader consequences of a warming environment on the life cycle of pathogens and the geographical spread of insect-borne diseases.

Thus, G20 Member States are presented with a picture of outbreak frequency and its drivers as a list of accepted facts.

Statements of WHO, the World Bank and G20 HLIP have impact, as they are widely quoted as authoritative and impact international and national policy. As explored below, such bold assertions are also found in the peer-reviewed literature. This practice promotes the development of affirmation bias via “citation proximity” and “citation inertia”, where controversial interpretations of data can inadvertently become accepted as fact [16]. 

The growing pandemic prevention agenda, partnering science, public health and industry, is premised on these assertions. If a genuine misattribution bias regarding spillover risk and consequent pandemic risk is arising, this can distort public health policy with potentially far-reaching consequences on health outcomes. In this case, these international agencies state clearly that pandemic risk is increasing due to an increase in outbreak risk (considered exponential) and that this is heavily driven by spillover events related to anthropogenic impact on the environment.

## Assertions in Prominent Peer-Reviewed Publications

Claims made by international agencies reflect the prevailing literature in peer-reviewed journals. While the literature is broad, we review here key papers cited by the international agency reports above and secondary publications upon which these also base their arguments, and certain widely cited opinion pieces supporting these arguments. The intent is not a comprehensive review of the impact of anthropogenic change and spillovers on pandemic risk, but to explore how a narrative has developed that is poorly supported by the data on which it relies.

### Anthropogenic Change and Habitat Interfaces

The contact of humans with wildlife at habitat interfaces, or ecotones, is a widely quoted and logical area of concern for the spillover of zoonotic diseases. Daszak et al., [17] in a 2001 opinion piece in *Acta Tropica*, set a tone that is repeated widely in subsequent publications. Discussing anthropogenic environmental change and its impact on the emergence of infectious diseases in wildlife, specifically the relatively low mortality diseases caused by Nipah Virus, Hendra viruses and West Nile Virus, they claim:

Since human environmental changes are largely responsible for their [zoonotic infectious disease] emergence, the threats wildlife EIDs [emerging infectious diseases] pose to biodiversity and human health represent yet another consequence of anthropogenic influence on ecosystems.

The paper does not present evidence for the assertion that humans are “largely responsible”, but justifies their claim by citing Mahy & Brown (2000): [18]

…the continued expansion of human populations brings us into contact with a large pool of known and unknown zoonotic pathogens.

However, Mahy and Brown’s paper is another general review of newly- and long-recognized diseases, ranging from influenza and HIV to less common examples including Nipah Virus and Ehrlichiosis. Thus, the relationship between environmental encroachment and increasing zoonoses is not directly tested nor the primary focus. In fact, Daszak et al. only find support for their assertion in the Mahy and Brown abstract, which states:

The increasing proximity of human and animal populations has led to the emergence of, or increase in, bacterial zoonoses such as plague, leptospirosis and ehrlichiosis.

The authors poorly support this statement in the main text. Plague (*Yersinia pestis*) is the widely assumed cause of the Black Death in Europe and subsequent major outbreaks [19]. Having previously caused devastating mortality, it is now confined to small outbreaks despite a rapidly increasing human population. Leptospirosis is found in many animal species, and its symptoms in humans are relatively non-specific [20]. Ehrlichiosis is uncommon and clinically non-specific, gained through bites of certain tick species. It seems reasonable to assume these diseases may have been a problem for centuries, but previously poorly distinguishable from clinically similar diseases.

Mahy and Brown note influenza as “continuing” to pose a risk. This is unsurprising from a virus group with proclivity to mutate and recombine. The only probable newly emerged disease raised by these authors is HIV/AIDS, which they state emerged in the 1980s, though its origin is now thought to have been several decades earlier [21]. This new understanding is significant, as it indicates that HIV/AIDS emerged in a time when human population was lower, and environmental change was proceeding at a very different pace.

Similarly to Daszak et al., [17] Despommier et al. [22] discuss ‘ecotones’, denoting the boundary between ecological systems, as areas where spillover of pathogens from one species to another (e.g. wildlife to humans) is likely to occur. They claim this is driving a “global trend of increasing EIDs [emerging infectious diseases].” To explain their hypothesis, the authors give the example of yellow fever, which is maintained through infection of monkeys in the forest canopy, but has caused human outbreaks as:

…human settlement has encroached along the forest fringes, and whose proximal habitations or plantations (e.g., banana) may support domestic and peridomestic Aedes spp. of mosquitoes.

This statement supports the authors’ contention if two implied assumptions are true; that human settlement is new or increasing along forest fringes (so this is a new risk), and if yellow fever is increasing. Regarding the claim of ‘encroachment on forest fringes’, the assumption that humans now have more interaction with forests because of increasing human population is often stated but is questionable. There are few tropical forest areas where humans have not traditionally lived, sometimes as hunter gatherers within the forest but often in fringe areas in slash and burn agriculture or as settled farmers. Deforestation rates have remained high, though decreasing, in recent decades [23], and the relationship between deforestation and disease transmission is complex (see discussion on Gottdenker et al. (2–14) discussed later) [11]. 

Regarding tends in yellow fever incidence, there was a far greater burden a century or more ago, both in the Americas where it was introduced through shipping, and in its endemic African ecological zone [24]. Yellow fever prevalence is therefore indicative of factors other than human population growth and modern anthropogenic change.

### Deforestation, Outbreak Risk and the Potential of Forest Restoration

Deforestation is also proposed as a major driver of disease emergence. Dobson et al. (2020) in Science [25] (cited in Rohr et al. below) began an opinion piece with:

For a century, two new viruses per year have spilled from their natural hosts into humans.

The cited reference is Woolhouse et al. [26] This statement, setting the basis for the Dobson paper, is a vital misrepresentation of what Woolhouse and co-authors actually wrote. Woolhouse et al. discuss the discovery (not emergence) of viruses that cause human disease, starting with the Yellow Fever virus in 1901 (after the identification of the tobacco mosaic virus in 1892 and the virus causing foot and mouth disease in cattle in 1898). They note that:

…New species of human virus are still being identified, at a rate of three or four per year.

**Fig. 1:**
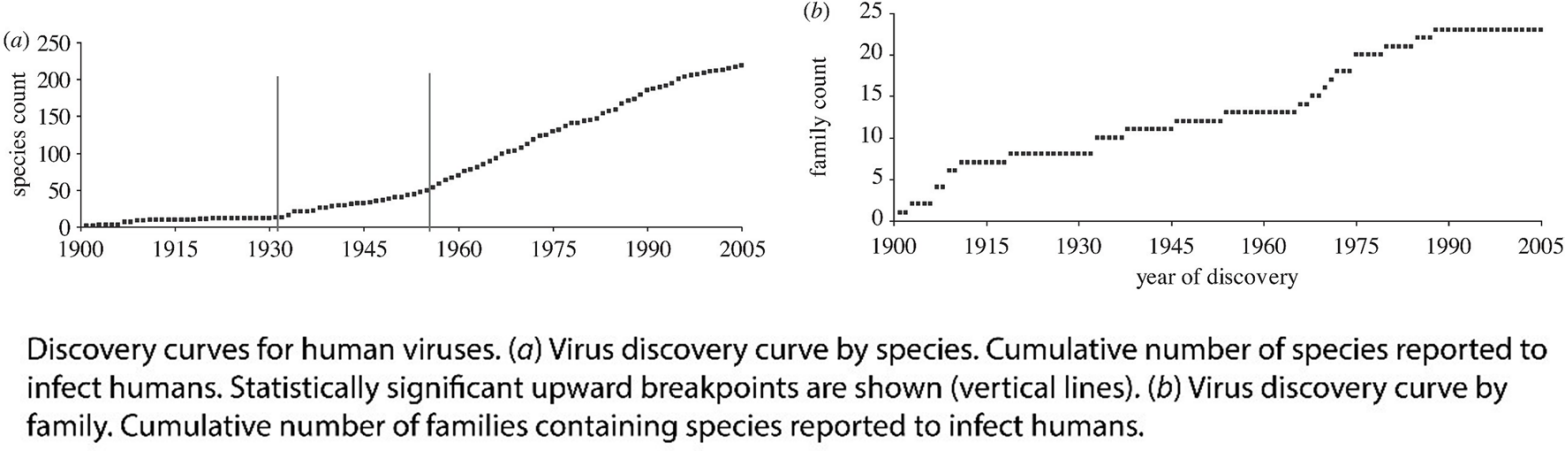
Rate of discovery of virus species and families that act as human pathogens, from Woolhouse et al. (2012).https://royalsocietypublishing.org/doi/10.1098/rstb.2011.0354

Identification started slowly just after 1900 and is now slowing again (Fig. 1). The authors note this flattening could indicate that the total number of viruses is bounded, or it could be another artefact of the techniques used to find them. The apparent addition of new species is at least partly an artefact of technological and research progress, a situation demonstrated by Rosenburg et al. (2013) where a clear acceleration in arbovirus discovery coincided with specific funding for the same in Africa and Asia from the Rockefeller Foundation, reducing as the funded program wound down [9]. Rosenburg, consistent with Woolhouse and colleagues, found pathogenic virus identification flattening after a peak in 1959–1969 – not a pattern suggestive of a rapidly increasing rate of new virus emergence.

Dobson et al. [25] go on:

The clear link between deforestation and virus emergence suggests that a major effort to retain intact forest cover would have a large return on investment even if its only benefit was to reduce virus emergence events.

This statement and the overall contention of the paper leads to the conclusion that $22 billion to $31 billion per year, spent to reduce deforestation, would produce a return on investment in terms of health outcomes. Yet, the entire premise depends on there being a “clear link” between continuing deforestation and overall disease burden.

The justification provided for this link in the Dobson paper includes contact with bat species acting as reservoir hosts of Ebola virus, Nipah Virus, SARS and SARS-CoV-2 [25]. The origin of SARS-CoV-2 remains contentious, since there is increasing evidence suggesting the involvement of human intervention through gain of function research [27–30], rather than a natural spillover event.

The other examples add little weight to Dobson et al.’s argument. Ebola virus is confined to West and Central Africa. The mode of spillover, presumed from hunting infected apes or directly from the bat reservoir [31], may have occurred for as long as humans have lived and hunted in African forests. Numbers do not suggest a rapidly expanding problem. The largest outbreak in history, in 2014-15 [32], killed less than 12,000 people or the equivalent of 4 days of tuberculosis mortality [33]. Nipah virus and Hanta virus disease each have less than 1000 recorded deaths globally, in geographically-confined areas [34, 35]. Regardless, Dobson and co-authors contend that these outbreaks are driven by increasing human disturbance to the bat’s forest habitat:

Fruit bats (Pteropodidae in the Old World, the genus Artibeus in the New World) are more likely to feed near human settlements when their forest habitats are disturbed; this has been a key factor in viral emergence in West Africa, Malaysia, Bangladesh, and Australia.

Three citations are provided in support of this statement. The first, Plowright et al., [36] is an opinion article proposing a model to guide understanding of zoonotic spillovers, and mentions only one potentially bat-associated disease; Ebola. Secondly, Olivero et al. [37] found an association between Ebola outbreaks and recent (within 2 years) forest loss, and a moderate association with human population density.

Pulliam et al. (2012), the third citation, assess the first recognized Nipah virus outbreak in Malaysia in 1999 [38]. However, rather than suggesting that forest fringe disturbance was a major driver, they note that the index farm was a mixed pig farm and mango plantation, thus attracting fruit bats from some considerable distance. The authors also note the very long (international) range of fruit bats. Thus, a wide-ranging bat vector was attracted to a major food source with traditional mixed agriculture. This is a quite different picture to local deforestation, and it is difficult to see how forest preservation, thus preserving roosting habitats for the primary host, would be a leading intervention.

Similar advocacy for forest preservation as a strategy to reduce disease outbreaks is provided by Rohr et al. (2019) in *Nature Sustainability.* [39] This article is influential, in the top 1% of cited articles across scientific journals [39]. Rohr and co-authors set the scene with their first line:

Infectious diseases are emerging at an unprecedented rate with significant impacts on global economies and public health.

The authors cite Jones et al. (2008) for this statement. Jones and co-authors assessed an extensive database of outbreaks, concluding contrary to Rohr et al.’s 2019 statement that outbreak emergence peaked in the 1980s, related to wide immunosuppression due to HIV, and had reduced in 2004 when their data set ended.

Yet, despite their mischaracterization of Jones et al., Rohr and co-authors go on to state that:

…reduced biodiversity that accompanies agricultural intensification can increase zoonotic disease emergence and can worsen already endemic diseases.

This statement cites three further sources; Jones et al. (2013) [40], Dobson et al. (2006) [41], and Cohen et al. (2016) [42], Jones et al. explain a more complex relationship in which reduction in biodiversity can either increase or decrease zoonotic disease emergence, as will be discussed later. Dobson et al., as discussed above, made these claims based on rather tenuous evidence regarding increased spillover events and confusing emergence of new viruses with their identification.

Cohen et al. (2016) [42], the third reference for Rohr’s 2019 statement, is interesting. Their paper opens with a definitive statement like that of Rohr et al.:

Humans presently are contributing to unprecedented rates of infectious disease emergence,….

The first source cited by Cohen to justify this statement is Jones et al. (2008) [43], which as explained above, presents disease outbreaks (including those of antimicrobial resistant strains) as peaking in the 1980s and then declining, contrary to Cohen et al.’s claim. The second citation is Rohr et al. (2011) [44]. This is the same first author as Rohr et al. (2019) and includes Dobson (above) among its co-authors. Rather than presenting evidence of an unprecedented rate of infectious disease emergence, this Rohr et al. 2011 paper in Nature Sustainability discusses the evidence gaps in existing claims linking climate change to an increase in outbreaks, concluding with the statement:

Although there should be genuine concern regarding future disease risk for humans and wildlife, we discourage alarmist claims and encourage rigor, open-mindedness and broad thinking regarding this crucial and interdisciplinary global issue.

This stands in interesting contrast to the opening statement in this 2011 paper: [44]

Global climate change and the unprecedented rate of disease emergence represent two of the most formidable ecological problems of our time.

The five citations used to qualify this opening statement from 2011 include four discussions specifically on climate change, and Jones et al. (2008) (discussed earlier) on disease emergence which is again poorly represented. Interestingly, the Rohr et al. (2011) paper actually argues extensively that climate change could either increase or decrease risk, a nuance that becomes lost in such definitive statements.

Thus, we see a pattern of assertive statements of rapidly rising disease risk with anthropogenic impacts on ecology driving it. These are cited heavily, resting largely on opinion, which is a poor substitute for evidence. More concerningly, there is a consistent trend of misrepresenting cited papers. This builds a picture of human-driven environmental change that ends with definitive statements in the reports and policy promotion of international health agencies. The 2019 recommendation from Rohr et al. that more care be taken has not been similarly repeated. A widely cited *Nature* article from 2022 exemplifying this pattern is discussed in the next section.

### A Highly Cited Paper Built on Shaky Ground

Vora et al. (2022), in a Comment article in *Nature* [10], lay out a case for investment of about $20 billion annually to prevent an increasing threat of pandemics originating from zoonotic spillover events.

The article opens with a statement that natural spillover events have:

probably triggered every viral pandemic that’s occurred since the start of the twentieth century….

**Fig. 2:**
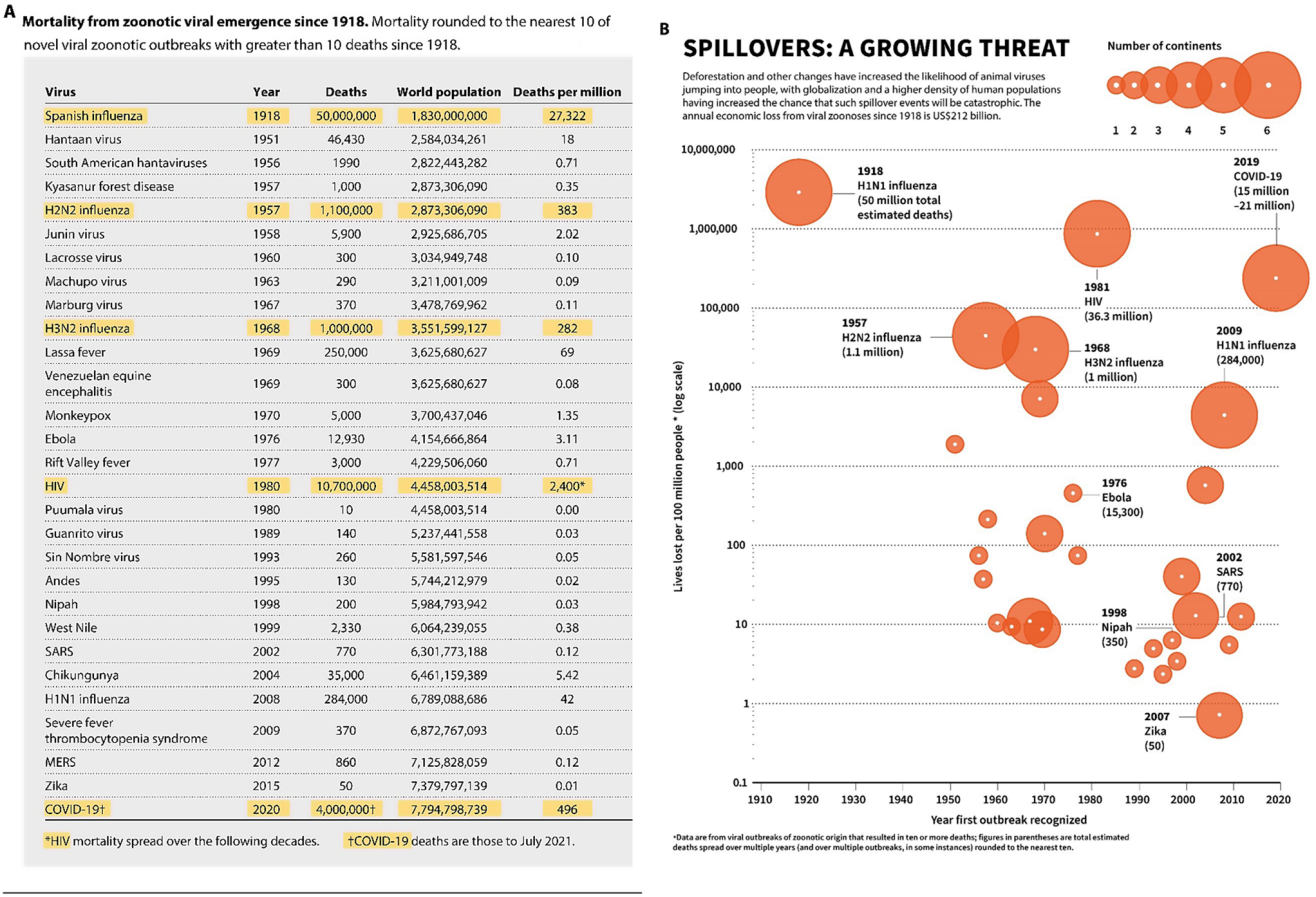
**A** Table 1 from Berstein et al.(2022) from which this figure appears to be deriver(highlights added). Highlights are added to emphasize the main drivers of mortality. **B** Figure from Vora et al.(2022) intended to demonstrate an increasing trend of mortality and geographical spread of pandemics

This claim cites Bernstein et al. (2022) [45], a paper which goes on to claim an annualized average mortality per year of 3.3 million, far higher than major endemic infectious diseases such as tuberculosis and an obvious justification for higher PPPR expenditure. Of relevance, Bernstein is last author on this Vora et al. paper. Discussed elsewhere [5], Bernstein et al. sought to assess outbreaks detected since 1918 that killed over 10 people to estimate annual mortality, proportional to current (2020) population size. They include the Spanish Flu from the pre-antibiotic era over a century ago, which greatly dominates the annualized burden, increasing it by almost a factor of ten as the authors acknowledge (Fig. 2A). There are no further outbreaks listed for 30 years until the 1950s. HIV, the second driver of mortality after Spanish Flu in Bernstein et al.’s analysis, is not commonly considered an acute outbreak, having potentially taken decades to become recognized after a probable spillover [21]. Covid-19, if accepting its widely disputed origin as a natural spillover event, is then the third largest burden event. Though the Covid-19 origin issue was widely debated in 2022 [46], both Bernstein et al. and Vora et al. ignore this major caveat in their cost and mortality estimates [5]. 

Covid-19 origins are fundamental to Vora et al.’s contention of financial return on proposed investment in PPPR, as PPPR as addressed in the 2024 IHR amendments and WHO’s draft Pandemic Agreement is focussed on detecting or preventing natural zoonotic outbreaks, as are related One Health investments. Vora’s relaince of Spanish Flu to achieve such high annual mortality is also problematic [45]. Secondary bacterial infection is widely thought to be the main cause of death during the Spanish Flu and is now treatable [47]. Thus basing average pandemic mortality on a Spanish Flu-like event is difficult to justify.

Lastly, Vora et al. claim that spillovers “*probably triggered every viral pandemic”*, which does not fit with Bernstein et al.’s outbreak list (Fig. 2A). This includes vector-borne pathogens such as Zika and Chikungunya viruses, where the main reservoir is human. While making little difference to overall burden, it does reinforce the impression of a lack of rigor.

Vora et al.’s return on investment assumptions rely on the assumption of anthropogenic impacts that can be greatly mitigated by the proposed interventions risk:

What’s more, an August 2021 analysis of disease outbreaks over the past four centuries indicates that the yearly probability of pandemics could increase several-fold in the coming decades, largely because of human-induced environmental changes.

Fortunately, for around US$20 billion per year, the likelihood of spillover could be greatly reduced.

The first sentence cites Marani et al. (2021) [48], and the second Dobson et al. (2020), discussed earlier [25]. A review of Marani et al. can be found elsewhere [5]. Briefly, Marani and co-authors estimate the frequency versus intensity of epidemics, based on detected pandemics since 1600. They do not directly find an increase in recent decades, but once more incorrectly cite Jones et al. (2008) for this [43]. Marani et al. conclude (in a 2022 correction) that major pandemics are actually rare, that a Covid-19-like event may be expected on average every 129 years, and a Spanish Flu-like event every 292 or 877 years (depending on assumptions regarding increased risk) [5]. These frequencies would reduce the annualized mortality of Bernstein et al. (2022) several fold, greatly reducing return on investment.

Dobson et al. (2020) base return on investment on major pandemics costing similarly to Covid-19 – estimated at about 5 trillion dollars [25]. However, this is predominantly the cost of the response, which was unprecedented and of disputed impact [49, 50]. Thus, Vora et al.’s opening statements that set the scene for their subsequent argument are in themselves extremely fragile, based on an assumption of the natural origin of Covid-19, the Covid-19 response being a typical response and cost, and far more frequent than their own sources suggest. It also relies on Spanish Flu-like events occurring with greater frequency than Marani et al. estimate, with antibiotics and modern medicine not affecting outcomes.

Vora et al., in their *Nature* Comment, further illustrate a perceived growing pandemic threat, and cost, as shown in Fig. 2B below. Though not stated, this appears to be derived from Table 1 of Bernstein et al. (2022) (Fig. 2A) [45], with the extent of geographical spread (number of continents) added.(Fig. 2B) Rather than showing a clear increase as claimed, this shows a relatively steady rate of outbreaks from the 1960s onward. If restricted to confirmed natural outbreaks – the subject of Vora et al.’s commentary – then removing Covid-19 will result in a reduction in mortality after 1990.

As further supportive evidence, Vora and co-authors note institutional support from the US Government, World Bank and WHO:

Over the past year, the administration of US President Joe Biden and two international panels (one established in 2020 by the WHO and the other in 2021 by the G20) have released guidance on how to improve approaches to pandemics. All recommendations released so far acknowledge spillover as the predominant cause of emerging infectious diseases.

The international panels, not directly referenced, appear to be the Independent Panel for Pandemic Preparedness and Response (IPPPR) established by WHO in response to World Health Assembly resolution 73.1. in 2020, and the High-Level Independent Panel (HLIP) of the G20 discussed earlier [2]. The IPPPR report is a collection of opinions in support of the WHO PPPR proposals [51]. It relies heavily on Covid-19 as a driver of the need for change, undermining Vora et al.’s and WHO’s messaging if SARS-CoV-2 origins did indeed involve laboratory processes [27, 28]. 

The G20 HLIP report, comprising Vora and co-author’s second source, reviewed in detail elsewhere [5], relies on two pieces of evidence to support its contention of increasing pandemic risk. The first, Annex D in the HLIP report of “major infectious disease outbreaks” from 2000 to 2020 includes less than 26,000 deaths globally if Covid-19 and the 2009 Swine Flu outbreak (with a lower mortality than seasonal influenza) are removed. The second are data from Metabiota Inc., showing an exponential increase in influenza outbreaks from near zero in 1960 (that logically are an artefact of improved testing and differentiation) and non-influenza outbreaks, which if Ebola is removed, actually show a decline in mortality from a peak with SARS in 2003 [5]. 

Thus, Vora et al., and by implication *Nature*, are accepting opinion as a basis for definitive statements on causality in a complex field, backed by citations that are very poorly supportive. This is important as (1) *Nature* is a widely trusted peer-reviewed source, and (2) the assessment advocates for proposed investments that are likely to result in considerable diversion of funds from areas of greater health burden [12]. 

## Recognizing a More Complicated Picture

A body of papers does exist, often sharing authorship with papers reviewed above, that paint a very different picture. Prominent among these is a review by Gottdenker et al. (2014) in EcoHealth [11], on the relationship between anthropogenic land use change and infectious disease. The authors reviewed 305 articles on the subject, noting a rapid upswing in publication since the year 2000 and finding 56.9% favouring an increase in infectious disease rates associated with land use changes, 10.3% noting a reduction, and 30.4% noting a variable response.

Gottdenker and co-authors note the possibility of various sources of publication bias. The increase in publication rate likely reflects an increase in funding. The concern, as always in scientific research, is that funder interest drives conclusions, as these impact the probability of further funding. Publishers may also prefer findings that fit a popular narrative and so promote readership. This would explain some of the simplistic, dogmatic statements included in papers discussed earlier. The authors also suggest the issue of anthropogenic change could be “value laden” - i.e. people want greater biodiversity to be good for health or want greater support for the preservation of natural habitats.

In discussing their more nuanced findings, Gottdenker et al. noted six possible ways in which anthropogenic change could influence infectious disease: [11]

1. Changes in vector habitats (niche),
2. Changes in host-vector composition,
3. Changes in spatial distribution of species (hosts or vectors),
4. Changes in species behaviour and movement (hosts, vectors, and interacting species),
5. Changes in socio-economic status of the human population, and.
6. Changes in stress levels and immune status.

These factors could influence the rate of disease outbreaks in either direction. An example of the complexity, and lack of predictability, can be found in malaria. Gottdenker et al. give an example of the Amazon, though the same situation can be found in Southeast Asia. Forest-dwelling vectors spread malaria to forest workers, which can cause an increase in transmission when there is heavy traffic at the forest fringes due to logging or land clearing. As agriculture becomes predominant and forest areas are eliminated, malaria declines. In Africa, as Gottdenker notes, development of rice paddies has increased local vector species habitats and thereby malaria, but the presence of grazing animals has then reduced transmission as some vectors preferentially feed on them. Enhanced prosperity associated with otherwise increasing vector habitats (e.g. rice paddies) may also lead to an overall reduction, outweighing the effect of the environmental changes involved [52]. 

This complexity and lack of predictability of anthropogenic change is highlighted in Jones et al. (2013) [40], mentioned earlier. The authors conducted a systematic review of the impact of agricultural intensification and environmental change on zoonotic disease in humans, determining:

We conclude that the rate of future zoonotic disease emergence or reemergence will be closely linked to the evolution of the agriculture–environment nexus. However, available research inadequately addresses the complexity and interrelatedness of environmental, biological, economic, and social dimensions of zoonotic pathogen emergence, which significantly limits our ability to predict, prevent, and respond to zoonotic disease emergence.

The potential complexity of such relationships is demonstrated by Dobson et al. (2006) [41]. Tick-borne encephalitis risk in the United States is dependent on a certain density of the yellow-necked mouse population. If there are too many mice, then there are insufficient ticks per mouse to maintain transmission of the virus between ticks. A very low mouse population reduces the food available to ticks and the tick population also drops. Higher densities of other mammals that provide a food source for ticks, but do not allow transmission of the virus between feeding ticks, further mitigates risk.

**Fig. 3:**
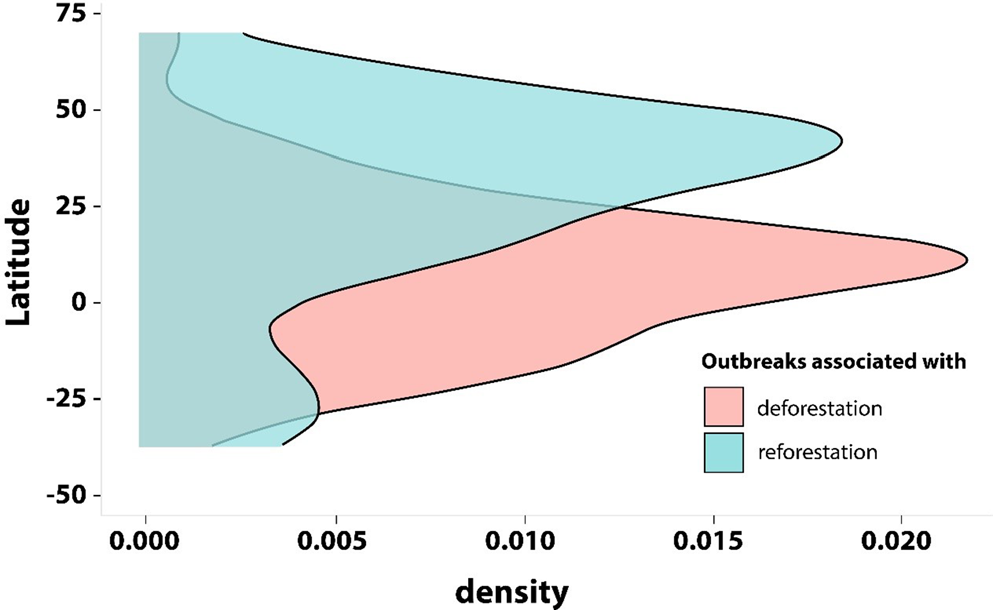
Association of outbreaks (by country) with reforestation and deforestation (Figure 5 in Morand & Lejaunie (2021) Source: https://www.frontiersin.org/articles/10.339/fvets.2021.661063/full

Morand and Lejauner (2021), cited in the G20 HLIP report, demonstrate such variability on a far larger scale in the association of deforestation on overall outbreak frequency at a country level (Fig. 3) [53]. Vector-borne disease and zoonotic outbreaks are associated with deforestation in the tropics, and reforestation at higher latitudes. This suggests vectors and pathogens with very different ecological requirements, but could also reflect a delayed ramping up of detection and recording technologies in generally lower income tropical countries with improved disease differentiation simply coinciding with deforestation in these countries.

Prior to 1960, most outbreaks were identified clinically or by light microscopy-based diagnosis. Basic methods for detection of antigens and antibodies (serology) were confined to well established laboratories. The succeeding 60 years, particularly after 1980, saw the advent of PCR, point of care antigen and serology testing, and genetic sequencing. This has been accompanied by developments in communications and recording. If we note that deforestation has largely been concentrated within the tropics over this period, particularly in the Amazon, areas of sub-Saharan Africa and South and Southeast Asia [54], then we would expect to see a corresponding association between deforestation and an increase in disease outbreaks, irrespective of causality.

Figure 3 would look generally similar if average income replaced latitude on the Y-axis. Are we viewing an effect of latitude, or an artefact of the development of diagnostic and surveillance capacity? As Gottdenker and co-authors imply, the true answer would probably be multifactorial.

**Fig. 4:**
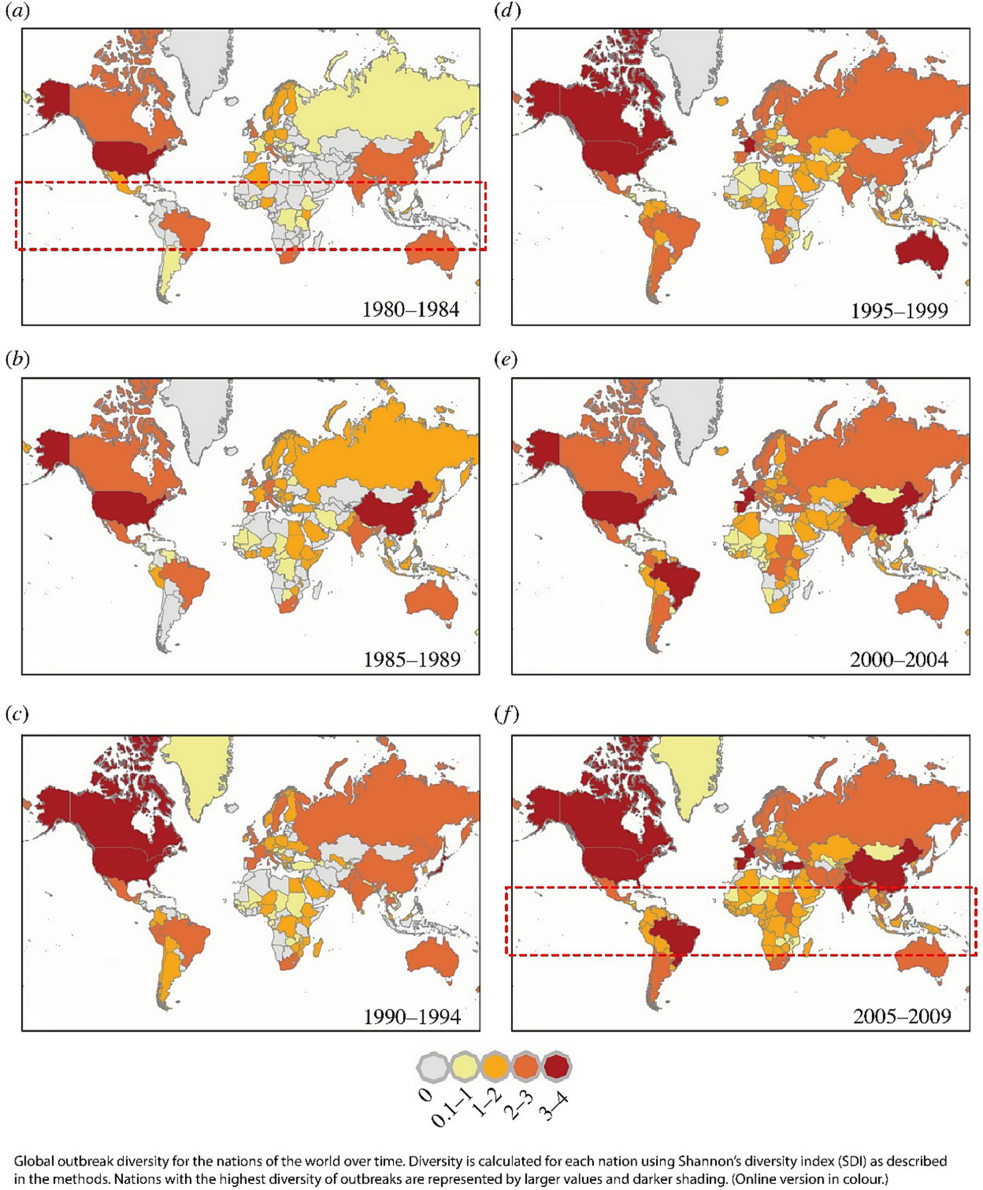
Outbreak reporting by country, 1984 to 2009, with the tropics highlighted in first and last maps. Adpated from Figure 2 Smith et al., 2014). Source: https://royalsocietypublishing.org/doi/10.1098/rsif.2014.0950

Smith et al. (2014) [55] (Fig. 4) is consistent with the capacity to detect being a major driver of reporting. They found outbreak reports were heavily dominated by higher income countries in the 1980s and early 1990s, with the notable exceptions of China and India where infrastructure was broadly more established. By 2009 reporting had become more uniform globally. This late increase in tropical countries is inconsistent with improvements in nutrition, sanitation and living conditions, which in higher income countries were associated with reduced infectious disease outbreaks [56, 57]. Unless there is a dramatic reduction in outbreaks in higher latitudes, which is not consistent with Fig. 4, we would also expect an overall increase in outbreak reporting globally. The ability to distinguish a pathogen or disease, and record it, confounds all disease tends.

The example of Nipah virus, cited by several papers reviewed here, serves as a good illustration. It was initially assumed to be Japanese encephalitis in its first recorded outbreak [34], before a newly recognized causative virus was isolated. However, its presence in multiple bat species across Asia and Africa, and its occurrence since in very limited and localized outbreaks, is also consistent with a long-standing but intermittent disease that was previously indistinguishable from the more common causes of encephalitis.

## Policy Implications

The PPPR program on which global public health is currently embarking, based on a perception of increasing risk of natural zoonotic spillovers driven by anthropogenic change, is unprecedented in scope and cost. As with any large public health program, allocation of resources to pandemic preparedness and response will divert funding from other public health and societal priorities. If natural spillover risk was actually constant or decreasing, consistent with infectious disease mortality overall [58], then this program is likely to cause net harm.

The extent of risk and trends of natural spillover events stated in WHO, World Bank and G20 reports promoting PPPR investment are not supported by the evidence sources they cite. If non-natural mechanisms underly risk, then quite different approaches would be used to prevent outbreaks based on laboratory biosecurity and restrictions on certain procedures. The massive investment in broad environmental surveillance and One Health under the IHR amendments and proposed Pandemic Agreement will do little to stop laboratory accidents. The continuing controversy regarding the origins of COVID-19 are important in this context, but irrespective of origin, the Covid-19 event is the only acute non-influenza outbreak to record over 15,000 deaths in the past century. To be persuasive in terms of increasing zoonotic risk, the purported underlying drivers (e.g. human-wildlife contact) must be shown to be increasing. Yet, as discussed above, their overall effects remain unclear.

With much of the reported increase in outbreak frequency consistent with an increase in ability to detect, distinguish and report, and with trends of reduction in frequency, and mortality, over the decade preceding COVID-19 [5, 59, 60], the justification for current PPPR investment proposals is further weakened. While increasing human populations are driving ecological change, the overall relationship of such change to spillover risk is complex, multivariant and difficult to quantify. The use of definitive throw-away statements of attribution and magnitude emanating from international health agencies and much of the published literature therefore provides a false impression of certainty.

There are several potential reasons for this tendency to reference opinion as if it is fact. The field has been relatively small, with authorship shared across many papers. This risks the development of a mechanism for circular referencing, reviewing and reinforcement of opinion, shielding claims from sceptical enquiry or external review. The increased interest of private-sector funders in public health institutions including WHO, and its emphasis on commodities in health responses, may deepen this echo chamber, inadvertently downgrading or ignoring contrary findings while emphasizing those studies that support further funding.

Furthermore, as noted by Gottdenker et al., [11, 60] an emphasis on the aspects of anthropogenic change that may drive spillovers over those that may mitigate them arises from habitat degradation being a “value-laden” issue. Preserving wilderness is widely perceived to be a global good, whilst environmental degradation, driving species to extinction and reducing diversity, is perceived as bad. Thus, although massive deforestation in Cambodia, Laos and Viet Nam have undoubtedly contributed to a reduction of malaria mortality, advocating for deforestation as an intervention would be widely perceived, at least by many in Western countries, as unacceptable. A preference among donors to support researchers expressing similar views (bureaucratic augmentation) may strengthen both value bias and congeniality bias further.

Lastly, journals have signalled a preference for a narrative that emphasizes environmental preservation, including its perceived impact on mitigating climate change [61]. This raises the potential for publication bias, also suggested by Gottdenker and co-authors. A paper that associates outbreaks with deforestation may have an easier passage through editors.

The PPPR approach for which WHO and partners are advocating, over $30 billion annually, with an additional $10 billion for One Health [3, 12], amounts to over ten times annual global spending on malaria [62] and over five times that on tuberculosis [33]. To justify PPPR investment and inevitable opportunity costs, it is imperative that the global health community develop a firmer evidence base. The literature suggests that interventions may need to be, at a minimum, highly tailored to local context. It is also plausible that many current policy directions, such as reforestation, could exacerbate risk in some contexts, whilst future ecological disruption can mitigate other risks. Further research would benefit our understanding of these processes but also create a more balanced and honest appraisal of the evidence already at hand and its role in policymaking. Based on their own evidence, the current claims of WHO and partner agencies in this area should be treated with healthy scepticism.

## Data Availability

No datasets were generated or analysed during the current study.
